# Strength enhancement of concrete using incinerated agricultural waste as supplementary cement materials

**DOI:** 10.1038/s41598-021-92017-1

**Published:** 2021-06-16

**Authors:** Teh Sabariah Binti Abd Manan, Nur Liyana Mohd Kamal, Salmia Beddu, Taimur Khan, Daud Mohamad, Agusril Syamsir, Zarina Itam, Hisyam Jusoh, Nur Amalina Nadiah Basri, Wan Hanna Melini Wan Mohtar, Mohamed Hasnain Isa, Nasir Shafiq, Amirrudin Ahmad, Nadiah Wan Rasdi

**Affiliations:** 1 Institute of Tropical Biodiversity and Sustainable Development, Universiti Malaysia Terengganu, 21030 Kuala Nerus, Terengganu Darul Iman Malaysia; 2 Department of Civil Engineering, Universiti Tenaga Nasional, Jalan Ikram-Uniten, 43000 Kajang, Selangor Darul Ehsan Malaysia; 3 Civil and Environmental Engineering Department, Faculty of Engineering, Universiti Teknologi PETRONAS, 32610 Seri Iskandar, Perak Darul Ridzuan Malaysia; 4 Geo TriTech, No. 17, Persiaran Perdana 15A, Pinji Perdana, 31500 Lahat, Perak Darul Ridzuan Malaysia; 5 Civil Engineering Department, Faculty of Engineering and Built Environment, Universiti Kebangsaan Malaysia, 43600 Bangi, Selangor Darul Ehsan Malaysia; 6 Civil Engineering Programme, Faculty of Engineering, Universiti Teknologi Brunei, Tungku Highway, Gadong, BE1410 Brunei Darussalam; 7 Faculty of Science and Marine Environment, Universiti Malaysia Terengganu, 21030 Kuala Nerus, Terengganu Darul Iman Malaysia; 8 Faculty of Fisheries and Food Science, Universiti Malaysia Terengganu, 21030 Kuala Nerus, Terengganu Darul Iman Malaysia

**Keywords:** Engineering, Materials science

## Abstract

The potassium (K) and sodium (Na) elements in banana are needed for hydration reaction that can enhance the strength properties of concrete. This research aims (a) to determine the material engineering properties of banana skin ash (BSA) and concrete containing BSA, (b) to measure the strength enhancement of concrete due to BSA, and (c) to identify optimal application of BSA as supplementary cement materials (SCM) in concrete. The BSA characterization were assessed through X-ray fluorescence (XRF) and Blaine’s air permeability. The workability, compressive strength, and microstructures of concrete containing BSA were analysed using slump test, universal testing machine (UTM) and scanning electron microscope (SEM). A total of 15 oxides and 19 non-oxides elements were identified in BSA with K (43.1%) the highest and Na was not detected. At 20 g of mass, the BSA had a higher bulk density (198.43 ± 0.00 cm^3^) than ordinary Portland cement (OPC) (36.32 ± 0.00 cm^3^) indicating availability of large surface area for water absorption. The concrete workability was reduced with the presence of BSA (0% BSA: > 100 mm, 1% BSA: 19 ± 1.0 mm, 2%: 15 ± 0.0 mm, 3% BSA: 10 ± 0.0 mm). The compressive strength increased with the number of curing days. The concrete microstructures were improved; interfacial transition zones (ITZ) decreased with an increase of BSA. The optimal percentage of BSA obtained was at 1.25%. The established model showed significant model terms (Sum of Squares = 260.60, F value = 69.84) with probability of 0.01% for the F-value to occur due to noise. The established model is useful for application in construction industries.

## Introduction

Agricultural waste is biomass produced by agriculture industries^1–4^. It is rich in fibres containing high amount of nutrients such as phosphorus and nitrogen, residues of pesticides and organic carbon^5,6^. Banana skin is waste biomass that can be adopted into various uses such as traditional medicine, livestock feeds, medium for mushroom cultivation and slow-release fertilizer for home garden plants^7^. It can be extracted into raw cellulose fibres as a reinforcing agent in composite polymers^8^. The banana skin can be processed into starch^9^. Incineration produces banana skin ash (BSA). Banana peel has been used for water treatment; especially for dye removal^10^ due to its physical adsorption capacity. BSA is comparable to other agricultural wastes^11^ for removal of various carcinogenic pollutants in water.

In material engineering properties, integration of natural fibres ash in concrete are continuously reported to be able to increase tensile^12^ and compressive strengths with improved modulus of elasticity in concrete higher than glass, steel and polymers^13,14^. Approximately 95 million tons agricultural waste of banana origin was produced since 2012^15^. It showed that, this waste has a high disposal rate throughout the year providing a sustainable supply for the construction industries. Similar to coal ash, this agriculture waste has low density, appropriate stiffness and satisfactory durability^16,17^, possesses pozzolanic properties making it a good candidate for partial replacement of cement, admixture or supplementary cement material (SCM) in concrete^15^; thus reducing construction costs. It has gained interest in civil construction and materials engineering fields ever since. Table 1 presents a comparison of strength enhancement using baby diapers polymers (BDP)^18^, coal bottom ash (CBA)^19^ and banana by-products from literatures (e.g. banana leaves ash (BLA)^15^, banana skin powder (BSP)^20^ and palm oil fuel ash (POFA)^20^, banana stem fibre (BSF)^14^) with the current research.

**Table 1:** Comparison studies on strength enhancement using baby diapers polymers, coal bottom ash and banana by-products from literatures.

Material	Type of concrete	Physico-chemical properties	28th days strength enhancement (MPa)	References
Baby diapers polymers (BDP)	High strength concrete	n.a	1% BDP: 61 10% BDP: 50	Mohamad et al.^18^
Coal bottom ash (CBA)	Normal concrete	n.a	10% CBA: 28 15% CBA: 33	Mohd Kamal et al.^19^
Banana leaves ash (BLA)	Normal concrete	Water absorption index, SEM, EDS, adherence resistance	5% BLA: 33 7.5% BLA: 36 10% BLA: 36	Kanning et al.^15^
Banana skin powder (BSP) and palm oil fuel ash (POFA)	Normal concrete	XRF, PSA	0.2% BSP: 22.3 0.8% BSP: 31.7 0.4% BSP + 15% POFA: 29.7 MPa 1% BSP + 15% POFA: 32.7 MPa	Mohamad et al.^21^
Banana stem fibre (BSF)	Normal concrete	Fibres linear density, single fibre tensile test, SEM	0.1% fibre: 28.9 0.2% fibre: 32.0 0.3% fibre: 29.7	Prakash et al.^14^
Banana skin ash (BSA)	Normal concrete	XRF, slump test, Blaine’s air permeability, SEM	Presented in Sub-chapter 3.2 in this research paper	Current research

*n.a.* not available.

The types of concrete used were high strength (> 40 MPa)^18^ and normal (20 to 40 MPa)^14,15,19,20^ concretes. The reported physicochemical properties from literatures were water absorption index^15^, scanning electron microscopy (SEM)^14,15^, energy dispersive X-ray analysis (EDS)^15^, adherence resistance^15^, X-ray fluorescence spectrometry (XRF)^20^, particle size analyser (PSA)^20^, fibre linear density^14^, and single fibre tensile test^14^. Current research reported physicochemical properties on XRF, Blaine’s air permeability, and SEM.

The application of BDP in high strength concrete produced strength enhancement from 50 (10% BDP) to 61 MPa (1% BDP)^18^. The integration of CBA in normal concrete produced strength enhancement from 28 (10% CBA) to 33 MPa (15% CBA)^19^. The addition of BLA in normal concrete yielded strength enhancement from 33 (5% BLA) to 36 MPa (7.5% and 10% BLA)^15^. The mixture of BSP in normal concrete produced strength enhancement from 22.3 (0.2% BSP) to 31.7 MPa (0.8% BSP)^20^. Meanwhile, the combination of 0.4% BSP + 15% POFA yielded 29.7 MPa and 1% BSP + 15% POFA yielded 32.7 MPa. The inclusion of BSF in normal concrete yielded strength enhancement to 28.9 MPa, 32.0 MPa and 29.7 MPa for 0.1%, 0.2% and 0.3% BSF accordingly.

Mohamad et al.^21^ reported that the use of BSA in conventional concrete improved the tensile modulus for optimum use by 5% as well as flexural strength but with considerably lesser compressive strength. It is noted that, different properties of various natural fibres are able to increase the strength properties of composites accordingly^12^. The application of banana leaf ash is technically feasible because not only does it improve the concrete performance but also contribute to reduction of cement utilization in construction industries; approximately up to 10% leading to construction cost reduction^15^. The building material cost reduction has been in good agreement with Prakash et al.^14^ who used banana stem fibres as an alternative to steel and artificial fibres to increase strength properties in mortar and concrete.

In addition, the current research presents observations on the microstructure improvement in the concrete after the integration of BSA via interfacial transition zone (ITZ). Response surface methodology (RSM) was used by Mohd Kamal et al.^19^ in their study to optimize the amount of CBA and strength enhancement in concrete. Thus, current research presents studies on the strength enhancement of concrete using incinerated agricultural waste namely, BSA as SCM. The optimization on the application of BSA as SCM in concrete was modelled via RSM.

## Methodology

### Material collection and preparation

**Figure 1:**
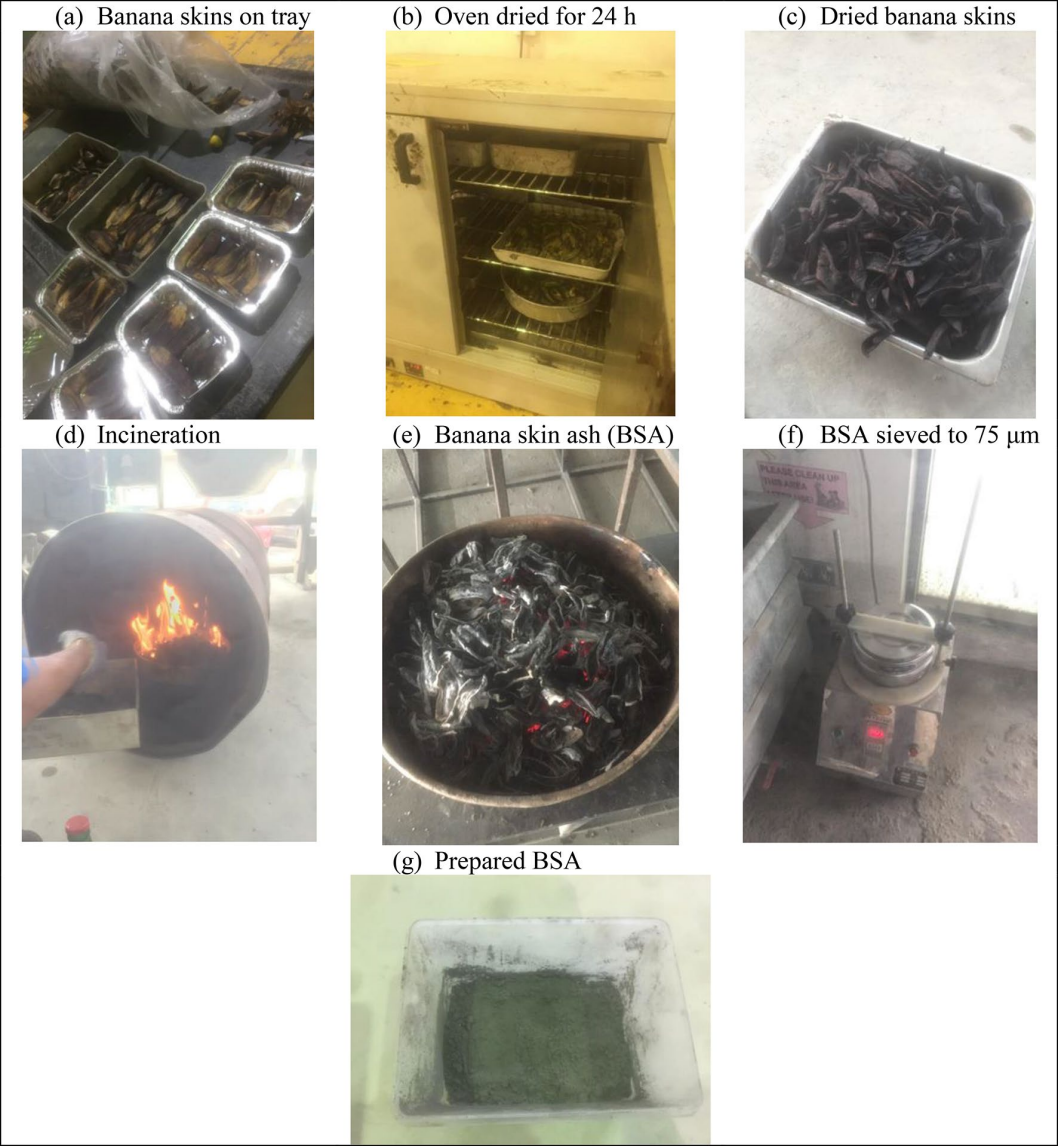
Preparation of BSA.

Banana skins locally called *pisang nipah* or Musa sp. were randomly collected from a local supermarket near Universiti Tenaga Nasional, rinsed several times with water, oven-dried (100 °C) for 24 h and incinerated at 300 °C for 30 min to produce banana skin ash (BSA). The resulting BSA was sieved to 75 μm sieve size. The preparation of BSA is shown in Fig. 1. Our study complies with relevant institutional, national, and international guidelines and legislation.

### BSA characterization

The material characterization was conducted based on X-ray fluorescence test (XRF), SHIMADZU CORPORATION, Tokyo, Japan) and workability test. The chemical composition of BSA samples was determined using XRF (RaynyEDX-700/800).

Blaine’s air permeability test or fineness test based on EN 196 ASTM C204 was conducted to obtain the fineness of BSA. The fineness of cement has a significant effect on the rate of hydration that will increase the concrete strength and the rate of heat evolution. Using Blaine’s apparatus, the specific surface for banana peel ash and cement was obtained.

### Engineering properties of concrete

#### Concrete workability

The slump test was conducted to assess the workability (or quality) of freshly made mix concrete focusing on the water-cement ratio based on ASTM C143 / C143M – 20 standards. The mix concrete contained 0 (control mix), 1 and 2% of BSA.

#### Concrete compressive strength

The compressive strength (MPa) of concrete was determined based on ASTM C39/C39M-21 using a universal testing machine (UTM). The curing days were ranged from 0 to 28 days. The structural composition of concrete was observed and recorded using scanning electron microscope (SEM) (ZEISS GEMINISEM 500, Oberkochen, Germany). The interfacial transition zone (ITZ) was also observed.

### Model optimization

Central composite design (CCD) with second order polynomial equation (Eq. 1) was used in response surface methodology (RSM) using the Design Expert ® software (Version 12) to assess the interrelation of process variables namely BSA composition in concrete and curing days with the response variable, compressive strength.

1 $$Y={\beta }_{0}+{\sum }_{i=1}^{k}{\beta }_{i}\cdot {x}_{i}+{\sum }_{i=1}^{k}{\beta }_{ii}\cdot {x}_{i}^{2}+{\sum }_{i\le j}^{k}\sum _{j}^{k}{\beta }_{ij}\cdot {x}_{i}\cdot {x}_{j}+\dots +e$$

where *Y *=  Predicted response variable, *β *=  Regression coefficient, *k *= Number of factors or process variables in the experiment, *e *=  Random error

The ranges of process variables and code factors are shown in Table 2. The error percentage between experimental and predicted values was evaluated using Eq. (2).

2 $${\text{Error}}~\left( \% \right) = ~\left| {\frac{{{\text{Experimental Value}} - {\text{Predicted Value}}}}{{{\text{ExperimentalValue}}}}~ \times ~100\% } \right|.$$

**Table 2:** Process variables and coded factors.

Process variables	Coded factors	Units	Low actual	High actual	Low coded	High coded
BSA	A	%	0	2.00	− 1	1
Curing days	B	Day	7	28	− 1	1

Pearson’s correlation coefficient (*r*) was used to measure linear association between two variables (Eq. 3) with the assumption that both variables are normally distributed. The correlation coefficient between variables can range from − 1 (shows negative linear correlation) to 0 (shows no linear relationship), to + 1 (shows positive linear correlation)^4,17,22,23^.

3 $$r = ~\frac{{~\mathop \sum \nolimits_{{i = 1}}^{n} ~\left( {x_{i} - ~\bar{x}} \right)\left( {y_{i} - ~\bar{y}} \right)~~}}{{\sqrt {~\mathop \sum \nolimits_{{i = 1}}^{n} ~\left( {x_{i} - ~\bar{x}} \right)^{2} ~\mathop \sum \nolimits_{{i = 1}}^{n} ~\left( {y_{i} - ~\bar{y}} \right)^{2} } }}$$

where, *r *= Correlation coefficient, *x*_*i*_ = values of the ‘x’ variable in a sample, $\bar{x}$ = mean of the values of the ‘x’ variable, *y*_*i*_ = values of the 'y' variable in a sample, *y* = mean of the values of the ‘y’ variable.

## Results and discussion

### BSA characterization

The XRF analysis (wt. %) for BSA, (a) percentages of oxide elements (%) and (b) percentages of non-oxides elements (%), is shown in Table 3. The Class F fly ash (FA) comes from burning anthracite, possesses pozzolanic property, with little or no cementitious value (silicon dioxide (SiO_2_) + aluminium oxide (Al_2_O_3_) + iron (III) oxide (FeO_3_) ≥ 80%); while Class C FA comes from lignite, and possesses both pozzolanic and cementitious properties (SiO_2_ + Al_2_0_3_ + FeO_3_ ≥ 50%)^24^. As compared to other materials, the silicon dioxide (SiO_2_) content of BSA (14.62%) was in the range close to OPC (20.6%). In the presence of heat during curing, the silicate-based material reacts with calcium hydroxide (Ca(OH)_2_) generated by hydrating cement to form compounds possessing cementitious properties of better high strength performance^25^. Meanwhile, the calcium oxide (CaO) (4.12%) content of BSA in the current study was comparable to FA (3.32%)^17,23^, bottom ash (BA) (7.37%)^17,23^ and class F FA (5%)^24^. The chemical compositions of BSA such as SiO_2_ (14.62%) + Al_2_O_3_ (0.26%) + FeO_3_ (0.21%) was 15.09%. Accordingly, BSA is a pozzolan but cannot be classified according to ASTM standards classification for coal ash pozzolans because the percentage composition were below the standards. Nevertheless, BSA can be classified as SCM that contributes to the properties of hardened concrete through hydraulic or pozzolanic activity. SCM are often added to concrete to make concrete mixtures more economical, reduce permeability, increase strength, or influence other concrete properties. Banana skin is high in potassium (K) content followed by manganese (Mn), sodium (Na), calcium (Ca), iron (Fe), bromine (Br), rubidium (Rb), strontium (Sr), zirconium (Zr) and niobium (Nb) (mg/g)^12,21^. The ‘K’ content in the BSA was 43.15%, the highest compared to other non-oxides elements. The non-oxide element Na was not detected. However; the chemical composition was found to be lower than that reported by Anwahange et al.^26^. The ‘K’ and ‘Na’ are crucial for hydration reaction in cement mortar for the enhancement of strength properties. These chemicals react with calcium hydroxide to form potassium hydroxide (KOH) and sodium hydroxide (NaOH), which will accumulate in the aqueous phase of the solution and contribute to a change in the composition of the pore fluid. This leads to a change in the pH of the medium and accelerates the hydration of the cement^27^.

**Table 3:** X-ray Fluorescence (XRF) analysis for BSA, (a) percentages of oxide elements (%) and (b) percentages of non-oxides element (%).

(a) Percentage of oxides element (%)	Chemical formula	Current research	Mohamad^21^	Kanning et al.^15^	Beddu et al.^17^ and Abd Manan et al.^23^			ASTM^24^	
		BSA	Banana skin powder	Banana leaves ash	Fly ash	Bottom ash	OPC	Class F fly ash	Class C fly ash
Silicon dioxide	SiO_2_	14.6222	55.98	48.7	46.8	50.75	20.6	52	35
Magnesium oxide	MgO	8.891	1.08		1.15	0.34	2.2	–	–
Sulphur trioxide	SO_3_	1.817	0.10		0.53	0.75	2.7	0.8	4.1
Calcium oxide	CaO	4.12	8.95		3.32	7.37	62.9	5	21
Potassium oxide	K_2_O	51.977	28.75		1.34	2.68	0.5	2.0	0.7
Aluminium oxide	Al_2_O_3_	0.256	2.71	2.6	18.41	17.91	4.4	23	18
Iron (II) oxide	Fe_2_O_3_	0.211	1.36	1.4	6.08	17.94	3.3	11	6
Phosphorus pentoxide	P_2_O_5_	4.728							
Zinc oxide	ZnO	4.711							
Manganese (II) oxide	MnO	0.728							
Dysprosium (III) oxide	Dy_2_O_3_	0.207							
Rubidium oxide	Rb_2_O	0.077							
Barium oxide	BaO	0.039							
Strontium oxide	SrO	0.038							
Praseodymium (III) oxide	Pr_2_O_3_	0.037							
Yttrium oxide	Y_2_O_3_	0.003							
Sodium oxide	Na_2_O	-		0.21					

(b) Percentage of non-oxides element (%)			Chemical formula			Current research			Anwanhange et al.^26^
Potassium			K			43.149			39.9
Oxygen			O			26.398			
Chlorine			Cl			7.4952			
Silicon			Si			6.8347			
Magnesium			Mg			5.3615			
Zinc			Zn			3.7847			
Calcium			Ca			2.9443			9.7
Phosphorus			P			2.0635			
Sulfur			S			0.72751			
Manganese			Mn			0.56355			38.3
Dysprosium			Dy			0.18079			
Iron			Fe			0.14779			0.3
Aluminium			Al			0.13534			
Rubidium			Rb			0.070671			0.11
Bromine			Br			0.042598			0.02
Barium			Ba			0.032296			
Strontium			Sr			0.032296			0.02
Praseodymium			Pr			0.031814			
Yttrium			Y			0.002491			
Sodium			Na			–			12.2
Zirconium			Zr			–			0.01
Niobium			Nb			–			0.01

Blaine’s air permeability results for BSA and OPC are shown in Table 4. OPC has high surface area (1470.8 ± 12.73) as compared to BSA (1091.2 ± 46.66). The OPC size or its fineness provides wider surface area for hydration that contributes to the hydration rate. It leads to the increasing in compressive strength development and heat evolution rate. It also increases the drying shrinkage of concrete^18,28,29^. The volume obtained for the BSA and OPC at 20 g of mass were 36.32 ± 0.00 cm^3^ and 198.43 ± 0.00 cm^3^ respectively. At the same mass, the BSA possessed a higher bulk density as compared to OPC. However, bulk density can be changed depending on the material’s handling (i.e. loose or compact)^30–33^. Time taken for the manometer liquid to drop from the second to the third level of the manometer during the test (second, s) were 8.33 ± 0.70 s (BSA) and 15.10 ± 0.26 s (OPC). The value measured during testing is the time required to pass a certain volume of air through a packed bed of solids with given size and porosity. The surface area is directly proportional to $\sqrt{t}$ ^34^. The higher the surface area, the shorter it takes for the time taken for air to flow through the compacted BSA and OPC beds.

**Table 4:** Blaine’s air permeability results for BSA and OPC.

Sample	Blaine air permeability test (fineness test)			Time taken for the manometer liquid to drop from the second to the third level of the manometer during the test (s)	Specific surface of sample
BSA	Mass (g)	0.90 ± 0.00	20.0 ± 0.00	8.33 ± 0.70	1091.12 ± 46.66
	Volume (cm^3^)	1.63 ± 0.00	36.32 ± 0.00		
OPC	Mass (g)	2.80 ± 0.00	20.0 ± 0.00	15.10 ± 0.26	1470.77 ± 12.73
	Volume (cm^3^)	8.93 ± 0.00	198.43 ± 0.00		

Results are presented in Mean ± Standard Deviation for triplicates.

### Engineering properties of concrete

#### Concrete workability

The percentages of BSA (%), slump (mm) and types of slump are shown in Table 5. The control mix concrete (0% BSA) has the highest slump of more than 100 mm and can be classified as collapse type of slump. It showed that the workability of the control mix is high. The BSA was used to substitute the OPC in the concrete mix at 1 and 2% BSA. The slump values were observed to be decreasing at increasing percentages of BSA. The slump values were 19 ± 1.0 mm (1% of BSA), and 15 ± 0.0 mm (2% of BSA). The workability of concrete was reduced with BSA addition.

**Table 5:** The percentages of BSA (%), slump (mm) and types of slumps.

Percentages of BSA (%)	Slump (mm)	Type of slumps
0	> 100	Collapse
1	19 ± 1.0	True slump
2	15 ± 0.0	True slump

Results are presented in mean ± standard deviation for duplicates.

Theoretically, the increase of more water content in the concrete mix will not only increase its workability but also may increase the potential of segregation between coarse aggregate particles, bleeding, drying shrinkage and cracking as well as decrease in the concrete strength and durability^35^. Nevertheless, ash is a good water absorbent^16,23,36^. Adding ash will reduce water content in the concrete mix and its workability. Although workability of the concrete was reduced, further test such as compressive strength and SEM were conducted to measure the strength and to observe the microstructure changes upon BSA addition as SCM.

#### Concrete compressive strength

**Figure 2:**
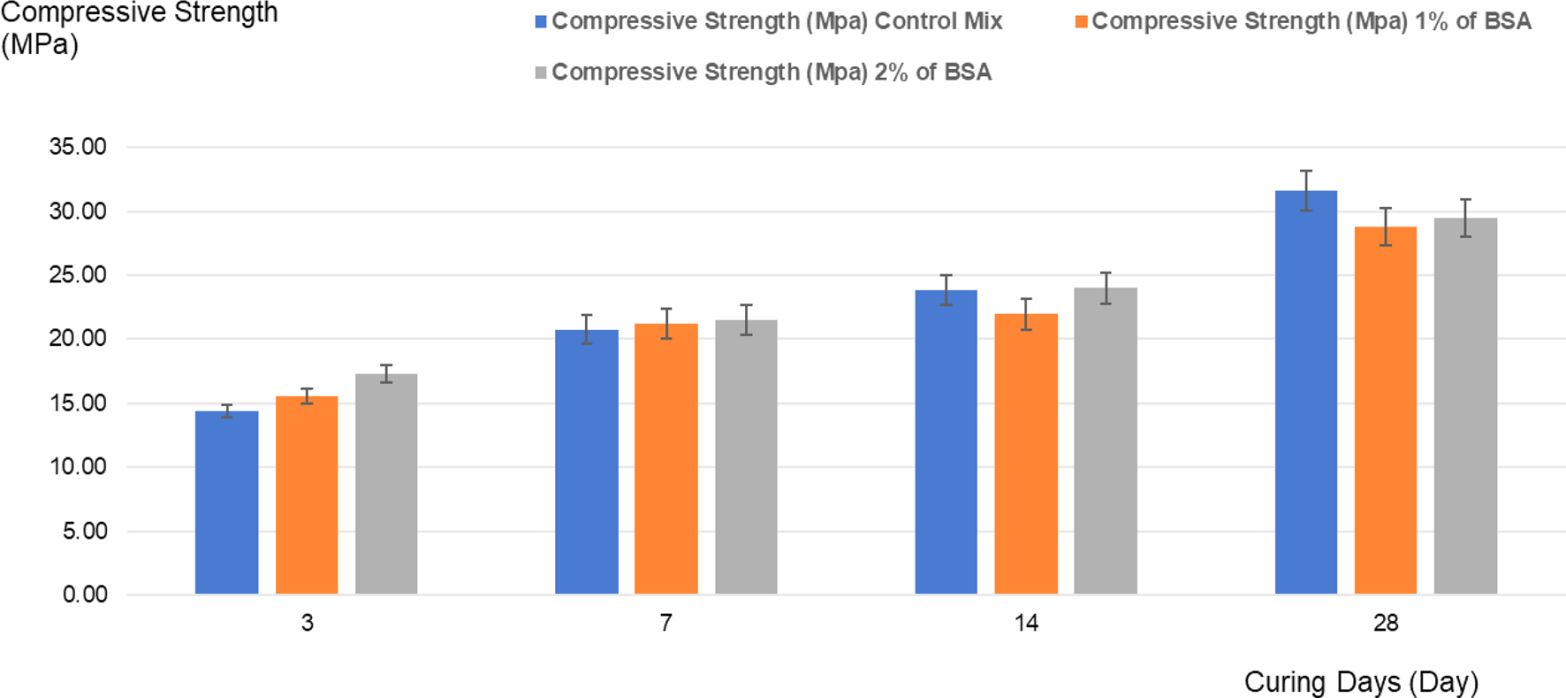
Comparison of compressive strength (MPa) of mortar (control mix, 1% of BSA and 2% of BSA) against curing days.

The percentages of BSA (%), ITZ (µm) and comparison of compressive strength (MPa) of mortar is shown in Table 6 and in Supplementary Material. The percentages of BSA against size of ITZ and compressive strength cover 0% (control mix), 1%, and 2% of BSA. As comparison of compressive strength (MPa) of mortar (0% (control mix), 1%, and 2% BSA) against curing days is shown in Fig. 2. The compressive strength for control was 14.42 ± 0.52 MPa (3 days), 20.76 ± 1.13 MPa (7 days), 23.86 ± 1.19 MPa (14 days) and 31.61 ± 1.60 MPa (28 days). The compressive strength for 1% BSA was 15.6 ± 0.58 MPa (3 days), 21.19 ± 1.16 MPa (7 days), 21.97 ± 1.20 MPa (14 days) and 28.77 ± 1.45 MPa (28 days). The compressive strength for 2% BSA was 17.3 ± 0.67 (3 days), 21.51 ± 1.18 (7 days), 24.00 ± 1.20 (14 days) and 29.50 ± 1.45 (28 days). The compressive strength was observed to increase with curing days duration to 28 days for all percentages of BSA studied.

**Table 6:** Percentages of BSA (%), ITZ (µm) and comparison of compressive strength (MPa) of mortar.

BSA (%)	ITZ (µm)	Compressive strength (MPa) of mortar against curing days			
		3	7	14	28
0	1.42 ± 0.38	14.42 ± 0.52	20.76 ± 1.13	23.86 ± 1.19	31.61 ± 1.60
1	1.32 ± 0.33	15.6 ± 0.58	21.19 ± 1.16	21.97 ± 1.20	28.77 ± 1.45
2	1.02 ± 0.15	17.3 ± 0.67	21.51 ± 1.18	24.00 ± 1.20	29.50 ± 1.45

Results are presented in mean ± standard deviation for triplicates.

**Figure 3:**
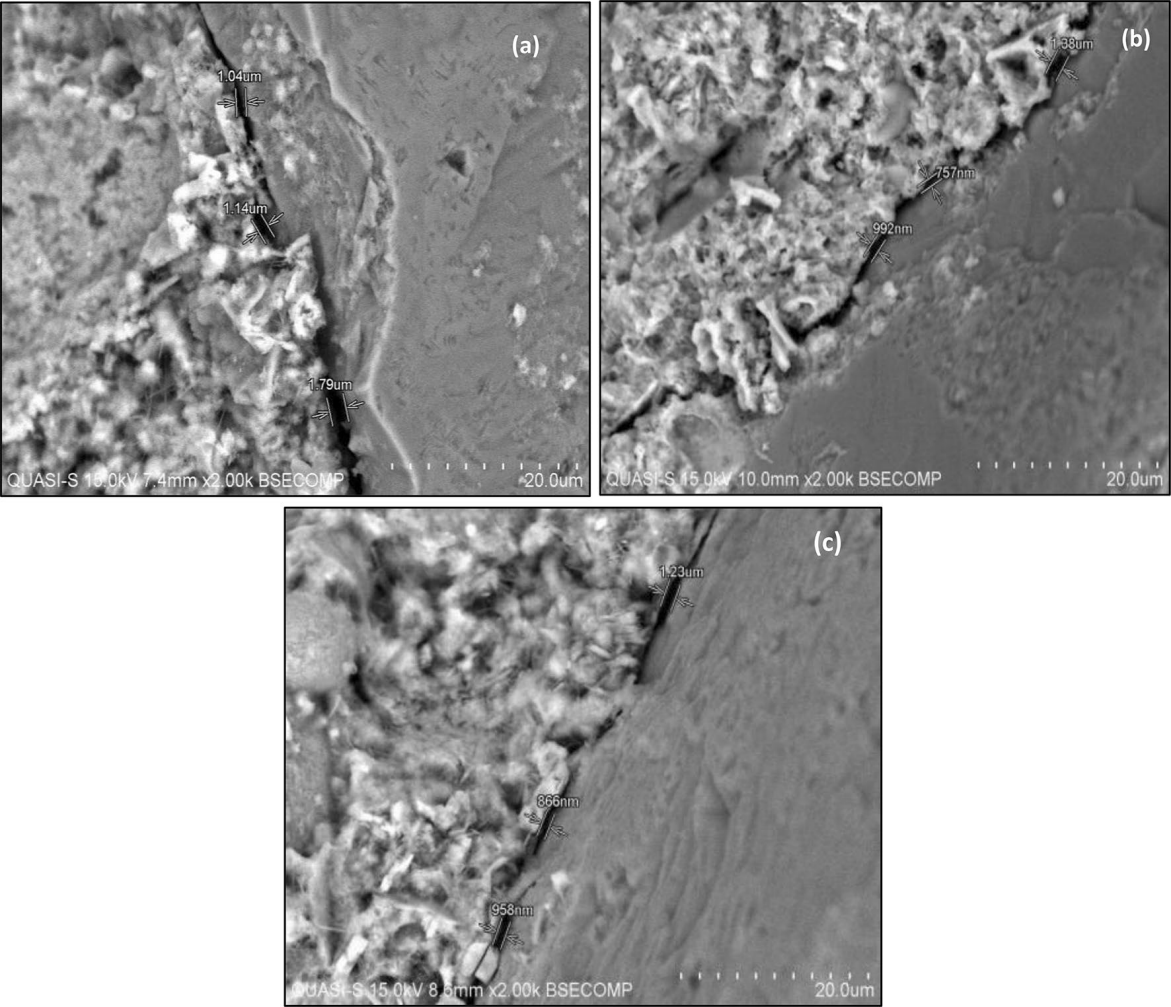
Three points of interfacial transition zone for (**a**) 0%, (**b**) 1% and (**c**) 2% of BSA at 2000 × of magnifications.

The sizes of ITZ obtained were 1.42 ± 0.38 µm (Control), 1.32 ± 0.33 µm (1% of BSA) and 1.02 ± 0.15 µm (2% of BSA) (Table 6). Figure 3 shows SEM images of ITZ in concrete containing BSA at 2000 × magnifications. Concrete contains aggregates and hydrated cement paste. A water cement ratio in a concrete develops around the aggregate particles during casting, bearing a different pattern of microstructure around the hydrated cement paste. The interfacial transition zone (ITZ) can be observed around the aggregate^37,38^. It has a strong influence on the mechanical properties of mortar and concrete^37–39^. BSA contains potassium that can enhance the properties of mortars in fresh and hardened conditions such as microstructure. The addition of BSA to mixture enhances the properties of the microstructure as the thickness of ITZ is decreased (Fig. 3).

### Model optimization

The 13 experimental runs pertaining to 2 process and 1 response variables were conducted for the RSM process optimization using CCD (Table 7). The process variables were percentage of BSA (%) and number of curing days (day). The ranges of process variables were 0 (control) to 2% BSA and 7 to 28 for curing days. The response variable was compressive strength (MPa). The analysis of variance (ANOVA) from the response surface quadratic model for compressive strength is shown in Table 8.

**Table 7:** CCD and responses results.

Run	Process variables		Response variable
	A (BSA, %)	B (Curing days, day)	Compressive strength (MPa)
1	1 (0)	17.5 (0)	23.1
2	1 (0)	17.5 (0)	23.1
3	1 (0)	17.5 (0)	23.1
4	0.4 (− 1)	17.5 (0)	23.86
5	2 (1)	28 (1)	29.5
6	0 (− 1)	7 (− 1)	20.76
7	1 (0)	17.5 (0)	23.1
8	2.4 (1)	17.5 (0)	25.57
9	2 (1)	7 (− 1)	21.51
10	1 (0)	2.65 (− 1)	15.6
11	0 (− 1)	28 (1)	31.61
12	1 (0)	17.5 (0)	23.1
13	1 (0)	32.3 (1)	33.27

**Table 8:** Anova for response surface quadratic model.

Source	*SS*	*DF*	*MS*	*F Value*	*Prob* > *F*	Indication
Quadratic model	260.60	5	52.12	69.84	< 0.0001	Significant
A-BSA	1.14	1	1.14	1.53	0.2557	Not significant
B-curing days	240.12	1	240.12	321.78	< 0.0001	Significant
AB	2.04	1	2.04	2.74	0.1418	Not significant
A^2^	13.28	1	13.28	17.80	0.0039	Significant
B^2^	4.28	1	4.28	5.73	0.0479	Significant
Residual	5.22	7	0.7462			
Lack of fit	5.22	3	1.74			
Pure error	0.0000	4	0.000			

**Figure 4:**
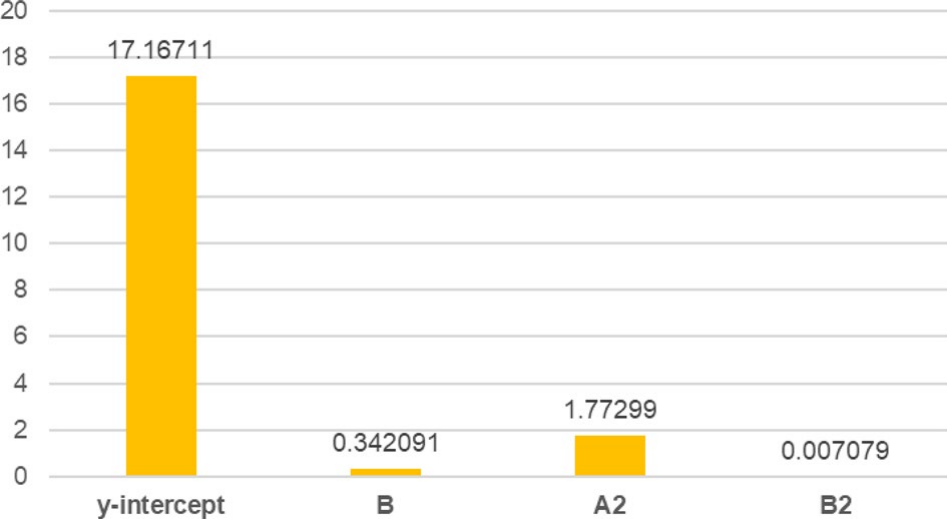
The β coefficient or regression coefficient of determination (*R*^*2*^) for quadratic model and model terms of actual factors.

The β coefficient or regression coefficient of determination (*R*^2^) for quadratic model and model terms for actual factors are 17.16711 (y-intercept), − 2.82036 (A), 0.342091 (B), − 0.068095 (AB), 1.77299 (A^2^) and 0.007079 (B^2^). Quadratic model and model terms B, A^2^, and B^2^ are significant (P-value < 0.05) (Fig. 4). The significant regression parameters of predictive models in terms of actual factors are shown in Eq. (4). The β coefficient for quadratic model and model terms of actual factors are shown in Fig. 4. Overall, the quadratic model is significant for the optimization purpose (Table 9). The *R*^*2*^ (0.9803) represents goodness of fit close to 1^40,41^.

4 $${\text{Compressive strength}}~\left( {{\text{MPa}}} \right) = 17.167 + 0.342091B + 1.77299A^{2} + ~0.007079B^{2}$$

**Table 9:** Fit summary results for response parameters.

Response	Significant model	Std. Dev	*R*^*2*^	*Adj. R*^*2*^	*Predicted R*^*2*^	*Adeq Precision*	*F-value*
Compressive strength (MPa)	Quadratic model	0.8638	0.9803	0.9663	0.7269	26.4046	69.84

**Figure 5:**
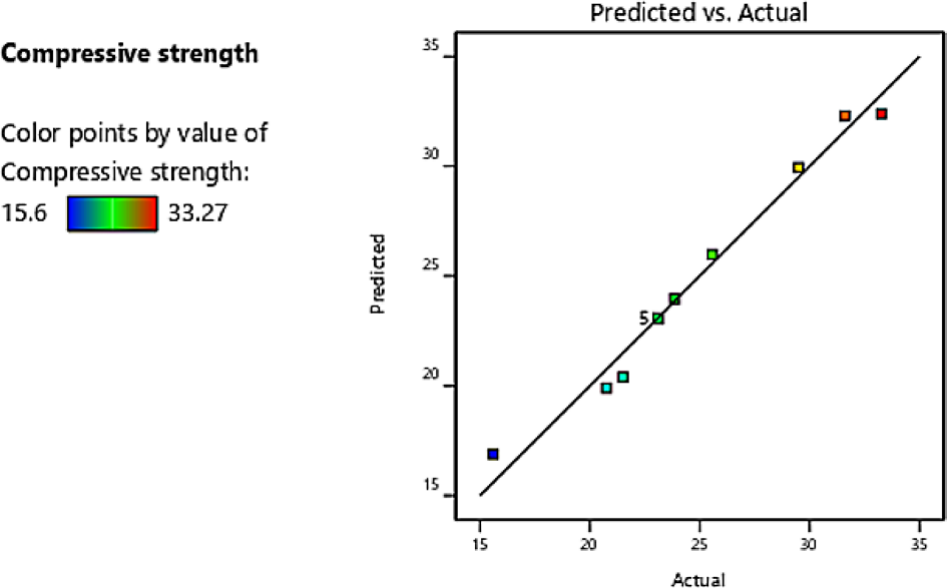
Predicted vs. actual values plot for compressive strength.

The patterns of predicted versus actual values plot for compressive strength is shown in Fig. 5. The scatterplots were in a straight line showing a linear relationship and the proposed model terms were sufficient, and constant variance assumption was verified.

**Figure 6:**
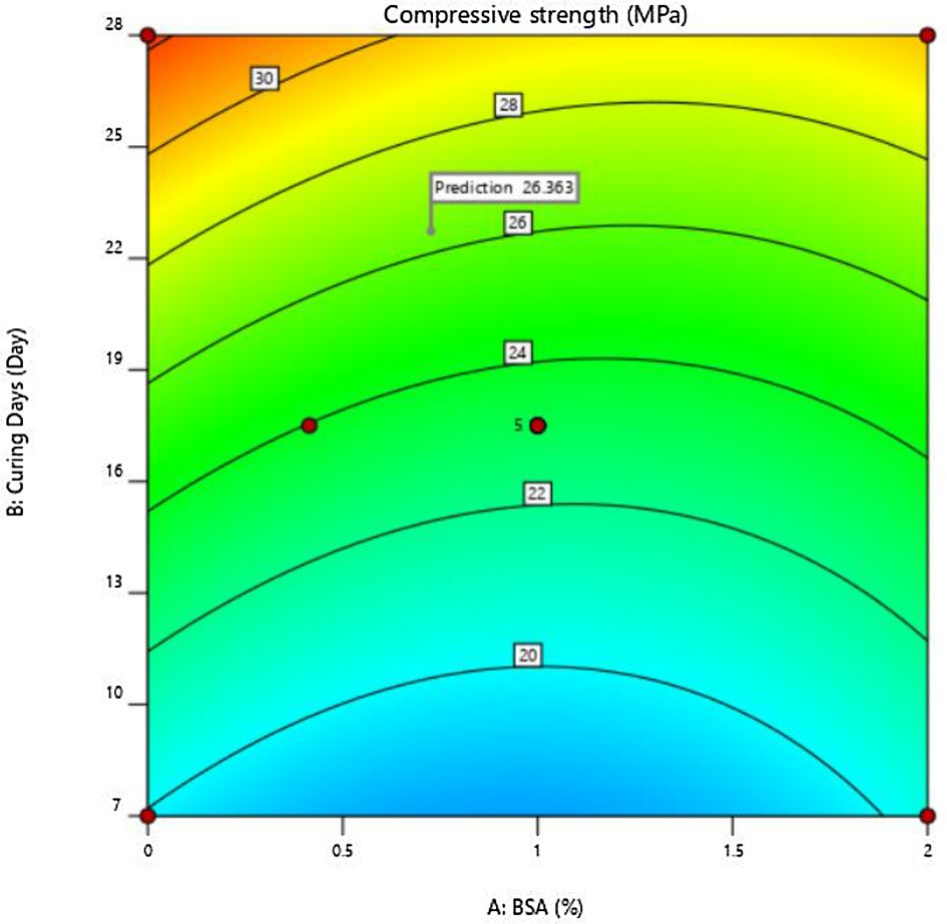
Contour plots for BSA (%), curing days (day) and compressive strength (MPa).

Figure 6 shows the contour plots for BSA (%), curing days (day) and compressive strength (MPa).

The compressive strength was found to increase with an increase in the number of curing days (7 to 28 days), which followed the usual strength development trend of concrete. The amount of BSA present in concrete also had an impact on its compressive strength. For a given days of curing, the compressive strength was highest at 1 to 1.5% BSA. Hence, 1.25% BSA has been adopted as optimum.

Table 10 shows validation of the optimised model. In engineering applications, a variability of 20% of error percentage is often acceptable^42,43^. As discussed previously, the optimal percentage of BSA is equivalent to 1% and can be observed from the minimal error percentage obtained such as 2.4%, 14.3%, and 18.5% for 7, 14 and 28 days of curing accordingly. Although the error percentage for control mix design is the lowest compared to 1% of BSA (4.1%, 2.4%, and 2.2% for 7, 14 and 28 days of curing accordingly), the microstructure was improved with concrete containing BSA. This improvement, reducing porosity and pore connectivity, leads to reduce the permeability and finally improve the durability^38^.

**Table 10:** Validation of optimised model.

Variables		Response: compressive strength (MPa)		Error percentage (%)
A: BSA (%)	B: curing days (day)	Experimental	Predicted	
0	7	20.76	19.90	4.1
1		21.19	21.70	2.4
2		21.51	27.00	25.5
0	14	23.86	23.30	2.4
1		21.97	25.10	14.3
2		24.00	30.50	27.1
0	28	31.61	32.30	2.2
1		28.77	34.10	18.5
2		29.50	39.40	33.6

**Figure 7:**
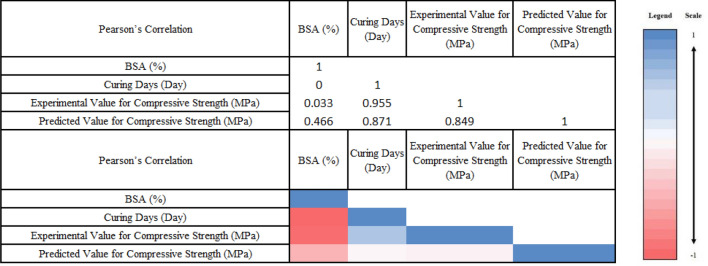
Pearson proximity matrices for BSA (%), curing days (day), experimental and predicted values for compressive strength (MPa).

The Pearson proximity matrices for BSA (%), curing days (day), experimental and predicted values for compressive strength (MPa) is shown in Fig. 7. The correlation coefficients obtained were highly positive between curing days (day) and experimental value for compressive strength (MPa) (R^2^: 0.955) as well as between curing days (day) and predicted value for compressive strength (MPa) (R^2^: 0.871). The experimental and predicted values were positively correlated with R^2^ equals to 0.849 indicating the optimised model is validated.

## Conclusion

Banana skin is an agriculture waste and BSA possesses pozzolanic property. BSA has high ‘K’ content crucial for hydration in concrete mix. The XRF analysed oxides and non-oxides elements of BSA that contribute to strength enhancement. Slump test showed that the workability of concrete reduced with addition of BSA. At the same mass of 20 g, BSA (36.32 ± 0.00 cm^3^) has a higher bulk density than OPC (198.43 ± 0.00 cm^3^) indicating large surface area for water absorption. The compressive strength of concrete increased with the number of curing days. The ITZ improved with the increased in BSA (control: 1.42 ± 0.38 µm, 1% BSA: 1.32 ± 0.33 µm, 2% BSA: 1.02 ± 0.15 µm). Overall, the optimal percentage of BSA was 1.25% at which the with the compressive strength was maximum for all curing days studied. The optimal number of curing days obtained was 28 days. The established model for strength enhancement of concrete using BSA as SCM was statistically significant and showed that it adequately represented the design space. The Pearson’s proximity matrices showed correlation between curing days and compressive strength (both experimental and predicted). The established model can assist in the application of BSA in construction industries.

## Supplementary Information

Supplementary Information.
