# Supplementary material for: Strength enhancement of concrete using incinerated agricultural waste as supplementary cement materials
Source: Sci Rep. 2021 Jun 16;11:12722. doi: 10.1038/s41598-021-92017-1 (PMC8209195; doi:10.1038/s41598-021-92017-1)

Universiti Tenaga Nasional

(Fakulti Kejuruteraan Awam)
Km 7, Jalan Kajang-Puchong
43003 Kajang, Selangor

Test Report (Compression)

(a) Control mix (0% BSA), 3 days

Test Date:04-10-2019
Test Time: 11:11:15

No.	Sample	Weight g	Length mm	Height mm	Width mm	Area mm²	Elastic modulus MPa	Stress N/mm²	Max. Load kN
1	3D CM S1	254	50	50	50	2500	1083.21	14.62	36.54
2	3D CM S2	253	50	50	50	2500	2112.46	14.42	36.04
3	3D CM S3	253	50	50	50	2500	2426.61	14.73	36.82

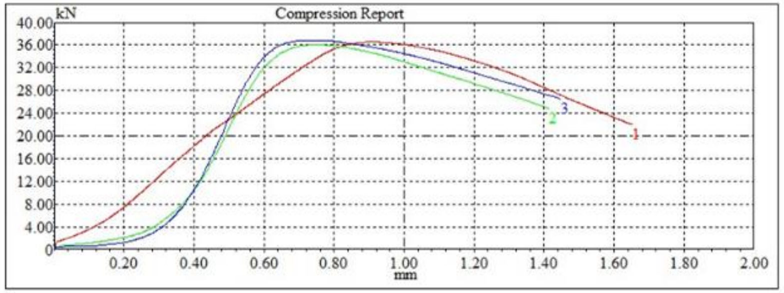

(b) Control mix (0% BSA), 7 days

Test Date:08-10-2019
Test Time: 9:22:00

No.	Sample	Weight g	Length mm	Height mm	Width mm	Area mm²	Elastic modulus MPa	Stress N/mm²	Max. Load kN
1	7D CM S1	254	50	50	50	2500	1184.24	20.68	51.71
2	7D CM S2	262	50	50	50	2500	3844.82	27.40	68.51
3	7D CM S3	258	50	50	50	2500	3117.10	20.83	52.07

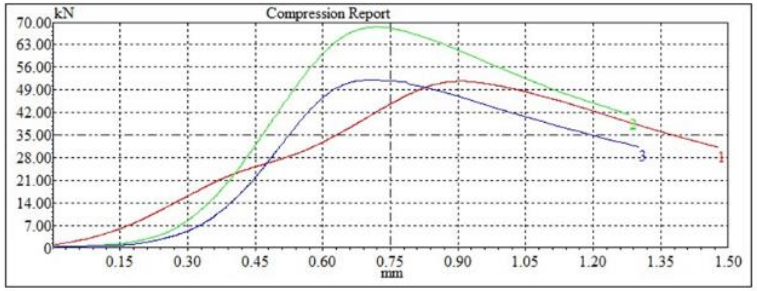

(c) Control mix (0% BSA), 14 days

Test Date:15-10-2019
Test Time: 9:24:22

No.	Sample	Weight g	Length mm	Height mm	Width mm	Area mm²	Elastic modulus MPa	Stress N/mm²	Max. Load kN
1	14D CM S1	253	50	50	50	2500	2290.03	23.86	59.66
2	14D CM S2	258	50	50	50	2500	3910.05	28.59	71.48
3	14D CM S3	270	50	50	50	2500	4119.91	31.78	79.46

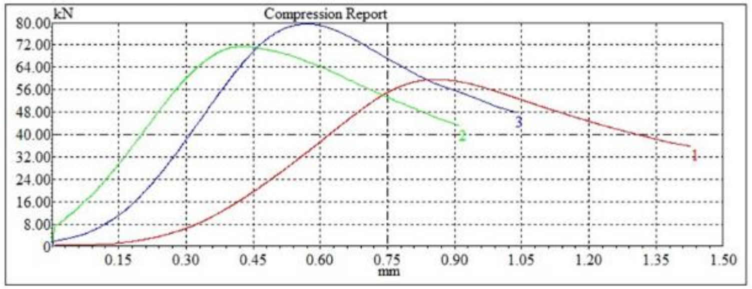

(d) Control mix (0% BSA), 28 days

Test Date:29-10-2019
Test Time: 9:31:12

No.	Sample	Weight g	Length mm	Height mm	Width mm	Area mm²	Elastic modulus MPa	Stress N/mm²	Max. Load kN
1	28D CM S1	263	50	50	50	2500	3873.41	33.70	84.26
2	28D CM S2	259	50	50	50	2500	3588.73	31.51	78.78
3	28D CM S3	259	50	50	50	2500	3986.17	31.71	79.28

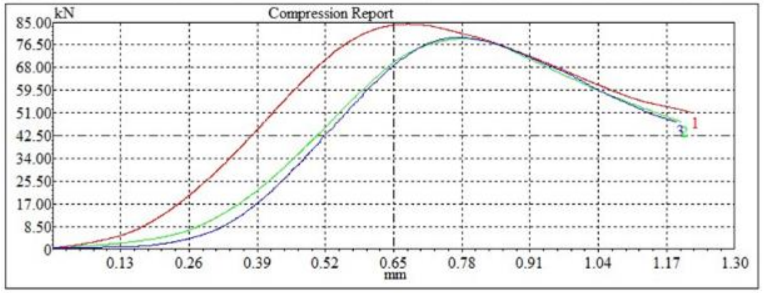

(a) Mix (1% BSA), 3 Days

Test Date:30-09-2019
Test Time: 10:19:51

No.	Sample	Weight g	Length mm	Height mm	Width mm	Area mm²	Elastic modulus MPa	Stress N/mm²	Max. Load kN
1	3D 1% S1	235	50	50	50	2500	1995.74	19.26	48.15
2	3D 1% S2	259190	50	50	50	2500	3363.77	20.55	51.37
3	3D 1% S3	251780	50	50	50	2500	2553.12	16.85	42.13

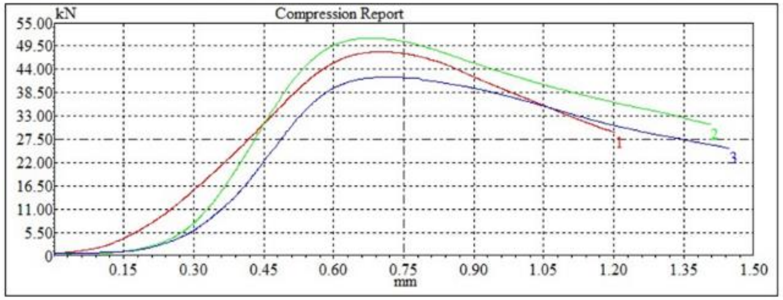

(b) Mix (1% BSA), 7 Days

Test Date:03-10-2019
Test Time: 10:40:57

No.	Sample	Weight g	Length mm	Height mm	Width mm	Area mm²	Elastic modulus MPa	Stress N/mm²	Max. Load kN
1	7d 1% S1	260	50	50	50	2500	2145.17	21.19	52.97
2	7d 1% S2	253	50	50	50	2500	3441.54	23.79	59.49
3	7d 1% S3	261	50	50	50	2500	3216.89	19.87	49.66

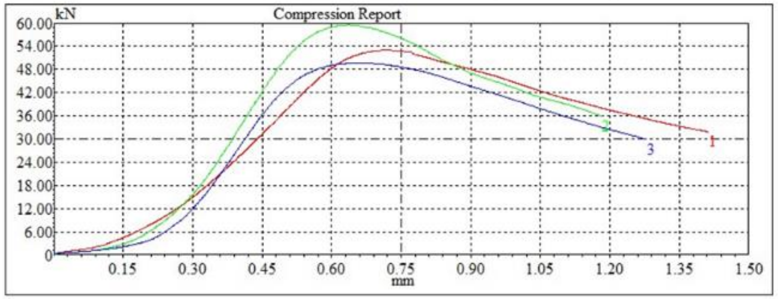

(c) Mix (1% BSA), 14 Days

Test Date:10-10-2019
Test Time: 10:20:45

No.	Sample	Weight g	Length mm	Height mm	Width mm	Area mm²	Elastic modulus MPa	Stress N/mm²	Max. Load kN
1	14D 1% S1	262	50	50	50	2500	4249.62	28.18	70.45
2	14D 1% S2	258	50	50	50	2500	9690.98	27.68	69.21
3	14D 1% S3	258	50	50	50	2500	528.41	15.75	39.36

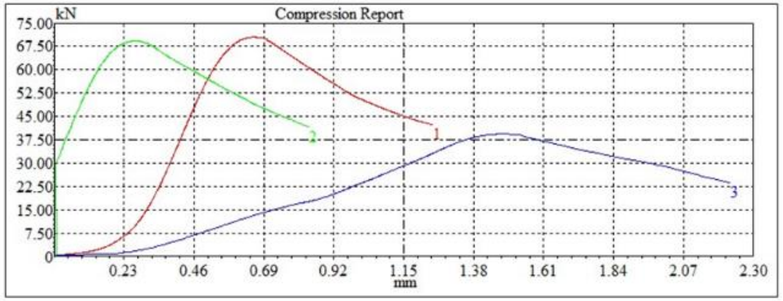

(d) Mix (1% BSA), 28 Days

Test Date:24-10-2019
Test Time: 9:22:12

No.	Sample	Weight g	Length mm	Height mm	Width mm	Area mm²	Elastic modulus MPa	Stress N/mm²	Max. Load kN
1	28D 1% S1	250	50	50	50	2500	2822.36	28.77	71.93
2	28D 1% S2	259	50	50	50	2500	3391.90	27.98	69.94
3	28D 1% S3	263	50	50	50	2500	2540.09	27.36	68.39

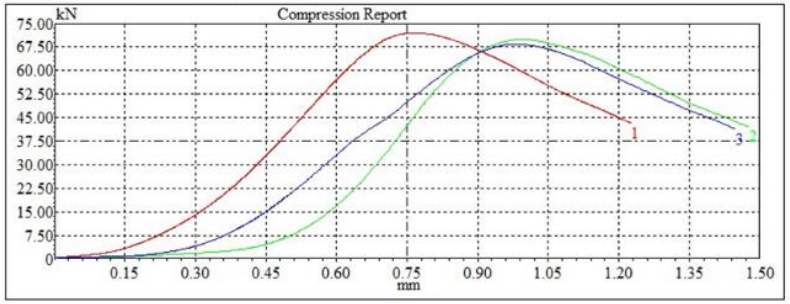

(a) Mix (2% BSA), 3 Days

Test Date:16-12-2019
Test Time: 9:41:37

No.	Sample	Weight g	Length mm	Height mm	Width mm	Area mm²	Elastic modulus MPa	Stress N/mm²	Max. Load kN
1	3D 2% S1	231	50	50	50	2500	493.53	13.31	33.28
2	3D 2% S2	237	50	50	50	2500	802.29	16.36	40.91
3	3D 2% S3	240	50	50	50	2500	738.90	16.49	41.23

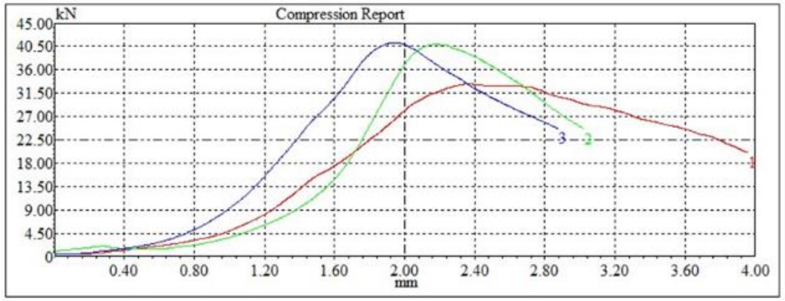

(b) Mix (2% BSA), 7 Days

Test Date:20-12-2019
Test Time: 9:25:31

No.	Sample	Weight g	Length mm	Height mm	Width mm	Area mm²	Elastic modulus MPa	Stress N/mm²	Max. Load kN
1	7D 2% S1	244	50	50	50	2500	1095.42	18.61	46.53
2	7D 2% S2	241	50	50	50	2500	1225.28	21.51	53.79
3	7D 2% S3	260	50	50	50	2500	802.76	13.39	33.48

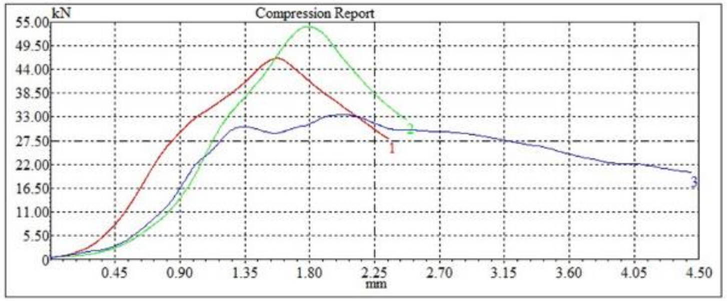

(c) Mix (2% BSA), 14 Days

Test Date:27-12-2019
Test Time: 11:37:40

No.	Sample	Weight g	Length mm	Height mm	Width mm	Area mm²	Elastic modulus MPa	Stress N/mm²	Max. Load kN
1	14D 2% S1	257	50	50	50	2500	1587.48	24.00	60.01
2	14D 2% S2	238	50	50	50	2500	1423.74	21.44	53.61
3	14D 2% S3	247	50	50	50	2500	1195.38	23.73	59.32

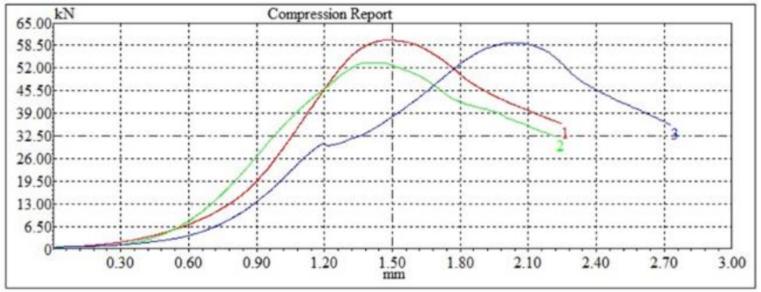

(d) Mix (2% BSA), 28 Days

Test Date:10-01-2020
Test Time: 11:13:27

No.	Sample	Weight g	Length mm	Height mm	Width mm	Area mm²	Elastic modulus MPa	Stress N/mm²	Max. Load kN
1	2% 28D S1	232	50	50	50	2500	1657.59	24.85	62.13
2	2% 28D S2	250	50	50	50	2500	1203.95	27.47	68.67
3	2% 28D S3	267	50	50	50	2500	2143.64	29.50	73.76

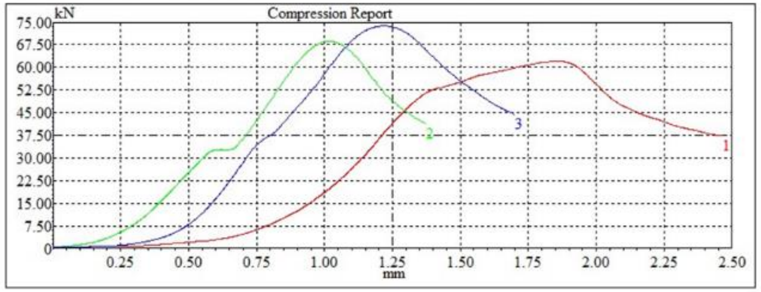

Supplement: Supplementary file 1 — Supplementary Information. [file 41598_2021_92017_MOESM1_ESM.pdf]
